# CYB561D2 up-regulation activates STAT3 to induce immunosuppression and aggression in gliomas

**DOI:** 10.1186/s12967-021-02987-z

**Published:** 2021-08-09

**Authors:** Bangbao Tao, Juanhong Shi, Shuai Shuai, Haiyan Zhou, Hongxia Zhang, Bin Li, Xiaoqiang Wang, Guohui Li, Hua He, Jun Zhong

**Affiliations:** 1 Department of Neurosurgery, Xinhua Hospital, Shanghai Jiaotong University, School of Medicine, 1665 Kongjiang Road, Shanghai, 200092 China; 2 Department of Pathology, Tongji Hospital, Shanghai Tongji University, No 389 Xincun Road, Shanghai, China; 3 Depatment of Oncology, Center Zhujiang Hospital, Southern Medical University, Guangzhou, 510280 Guangdong China; 4 Department of Pathology, Xiang-ya School of Medicine, Central South University, Changsha, 410013 China; 5 Department of Emergency, San Ai Tang Hospital, 74 Jing-Ning Road, Lanzhou, 730030 China; 6 Department of Anesthesiology, Xinhua Hospital, Shanghai Jiaotong University, School of Medicine, 1665 Kongjiang Road, Shanghai, 200092 China; 7 Department of Neurosurgery, Third Affiliated Hospital of Second Military Medical University, No 225 Changhai Road, Shanghai, 200438 China

**Keywords:** CYB561D2, STAT3, ROS, Gliomas, Immune checkpoint, Immunosuppression

## Abstract

**Background:**

Fine tuned balance of reactive oxygen species (ROS) is essential for tumor cells and tumor cells use immune checkpoints to evade attack form immunity system. However, it’s unclear whether there is any crosstalk between these two pathways. CYB561D2, an antioxidant protein, is part of 5-gene prognosis signature in gliomas and its involvement in gliomas is unknown. Here, we aim to provide a detailed characterization of CYB561D2 in gliomas.

**Methods:**

CYB561D2 expression was measured in clinical samples of gilomas and normal tissues. The effects of CYB561D2 on immunity related genes and tumor behaviors were investigated in glioma cell lines with various in vitro and in vivo assays.

**Results:**

CYB561D2 expression was enhanced in gliomas compared to control tissues. CYB561D2 up-regulation was associated with high grading of gliomas and short survival in patients. CYB561D2 expression was induced by H_2_O_2_ in glioma cell lines. CYB561D2 and its functional product ascorbate activated STAT3 dose-dependently. CYB561D2 over-expression increased PD-L1, CCL2 and TDO2 expression, and induced immunosuppression in co-cultured T cells. In in vitro assays, CYB561D2 knock-down suppressed cell growth, colony formation, migration and promoted apoptosis. In contrast, CYB561D2 over-expression reduced survival rate in intracranial glioma model and this effect could be blocked by dominant negative-STAT3. The CYB561D2 up-regulation and the positive association of CYB561D2 with PD-L1, CCL2 and TDO2 expression were cross-validated in open-access datasets.

**Conclusions:**

CYB561D2 up-regulation induces immunosuppression and aggression via activating STAT3 in gliomas and CYB561D2 mediates ROS-tumor immunity crosstalk.

**Supplementary Information:**

The online version contains supplementary material available at 10.1186/s12967-021-02987-z.

## Background

Gliomas are the most common primary brain tumors in adults and huge efforts have been made to investigate its pathogenesis [1]. Recent advance in deep sequencing has produced massive data on the genomes of various cancers including gliomas [2, 3]. Data mining of these large-scale sequencing projects could provide unprecedented opportunity to explore the complex genetic landscape of gliomas and reveal novel therapeutic targets. Indeed, using stringent and un-biased bioinformatics pipelines, we have identified a robust and reproducible gene signature which is an independent predictor for the survival of patients with gliomas in datasets with large sample size [4]. This gene signature includes HOXC10, LOC101928747, RPS4XP2, RPL36A and CYB561D2. Given their strong association with prognosis and high reproducibility, these genes may represent novel targets in gliomas.

Over-accumulation of reactive oxygen species (ROS) has been observed in most cancers [5]. Although ROS could facilitate the pathogenesis and progression of tumor, it also results in a compensatory response which up-regulates the expression of antioxidant proteins to detoxify ROS [6]. It suggests that fine tuned balance of intra-cellular ROS levels is essential for tumor cells. Interestingly, CYB561D2 is a member of Cytochromes b561 family and it’s a two-heme-containing cytochrome that catalyzes ascorbate-dependent trans-membrane ferric-chelate reduction [7]. Thus, CYB561D2 is involved in oxidation–reduction reaction [8, 9] and it’s very effective in the regeneration of ascorbate, a potent antioxidant [8].

Previous studies show that CYB561D2 is associated with lung cancer [10, 11]. However, the potential contribution of CYB561D2 to gliomas is totally unknown. Given the strong association of CYB561D2 with survival of patients and the important roles of CYB561D2 in the homeostasis of oxidation–reduction reaction, we hypothesize that CYB561D2 is potentially involved in the pathogenesis of gliomas. Here, we provide a detailed characterization of CYB561D2 in gliomas and our results suggest that CYB561D2 up-regulation induces immunosuppression and aggression via activating STAT3 in gliomas. It reveals a novel role of CYB561D2 in mediating the crosstalk between ROS and tumor immunity.

## Methods

### Clinical samples

The study was approved by the Review Boards of Xinhua Hospital (Shanghai, China) and conducted according to the principles of the Declaration of Helsinki. Written informed consent was obtained from each patient. A total of 35 cases of primary glioma tissues were collected in Xinhua Hospital. The cases were graded based on the 2007 WHO Classification criteria. All glioma cases were grouped as low grade (WHO I and II, n = 14) or high grade (WHO III and IV, n = 21). Seven normal brain samples were obtained from non-glioma patients undergoing brain surgery.

### Cell cultures

Cells were authenticated by STR profiling and tested for mycoplasma contamination. Glioma cell lines U87 and U251 were obtained from ATCC and maintained in Dulbecco’s Modified Essential Medium (DMEM) with 10% fetal bovine serum (FBS), 100 U/ml penicillin and 100 mg/ml streptomycin. Mouse T cells were isolated using Pan T cell isolation kit (130-095-130, Miltenyi Biotech).

### CYB561D2 over-expression and knock-down

The coding sequence of human CYB561D2 (NM_007022.4) was cloned into pcDNA3.1-B(−) plasmid. Plasmids were transfected at indicated dose using Lipofectamine 2000 for 48 h. For in vitro and in vivo assays of tumor behaviors, the coding sequence of human CYB561D2 was cloned into Lenti-EGFP vector and it was packaged and amplified in HEK293T cells.

For CYB561D2 knock-down, the short hairpin RNA (shRNA) targeting human CYB561D2 mRNA sequence (forward: TGCCTCACCAGCTTGGTCATTTTCAAGAGA AAT GACCAAGCTGGTGAGGCTTTTTTC; reverse: TCGAGAAAAAAGCCTCACCAGCTT GGTCATTTCTCTTGAAAATGACCAAGCTGGTGAGGCA) and its scrambled shRNA were constructed into the pLentiLox3.7 (pLL3.7) lentiviral vector.

To monitor the trafficking of STAT3, pEGFP-N1-STAT3 construct [12] in which human STAT3 is fused to GFP was transfected into cultured cells. The lenti-viral construct of dominant negative STAT3 mutant (Y705F-STAT3) [13] was used to block endogenous STAT3.

### In vitro and in vivo assays for tumor cell behaviors

In vitro assays were performed as previously described [14]. In brief, cell proliferation was measured by MTT assay, cell migration was measured by transwell assay, apoptosis is measured by RealTime-Glo™ Annexin V Apoptosis assay and Caspase-Glo® 3/7 assay. All experiment was biologically repeated for three times. Intracranial glioma model was established by stereotactical injection of U87 cells infected with lenti-virus (Control, CYB561D2, CYB561D2 + DN-STAT3; n = 13–15 per group) at MOI of 10 as previously described [15]. The care of nude mice was in accord with the animal welfare guidelines of Xinhua Hospital, Shanghai Jiaotong University.

### Quantification of intra-cellular ascorbate levels

Intra-cellular levels of ascorbate in cell lines were measured with commercial assay kit (ab65346, abcam) and the data were shown as fold changes relative to control group.

### Luciferase assay

The 1000 bp promoter of the human CYB561D2 was constructed into pGL4.10 vector. Luciferase reporter assays were performed as previously described [14].

### Quantitative real-time PCR

Quantitative real time-PCR (qPCR) was performed to measure the relative mRNA levels of CYB561D2 and immunosuppressive genes, as previously described [14]. The qRT-PCR primers were shown in Additional file 1: Table S1. Target gene expression was calculated using the ΔΔCt method.

### Western blot

RIPA lysis buffer was used to extract proteins from tissues or cell lines and protein samples (50 μg) were resolved by SDS-PAGE and then probed with indicated antibodies. CYB561D2, FAS ligand (FASLG), TGF-β2 (TGFB2), CD70, PD-L1, PD-L2, CCL2, TDO2, total STAT3, p-STAT3 (Tyr705) antibodies were from Sigma-Aldrich (SAB2500281), abcam (ab15285), abcam (ab36495), abcam (ab77868), abcam (ab205921), abcam (ab187662), abcam (ab186421), abcam (ab123403), CST (4904) and CST (9131), respectively. STAT3 inhibitor C188-9 was from selleckchem (S8605) [16]. The band density was analyzed with Image J and the density of target protein is normalized by Tubulin band. There are four biological replicates for each WB experiment.

### Immunohistochemistry

Immunostaining of tissue sections at 6 µm thickness with CYB561D2 antibody (Sigma-Aldrich, SAB2500281, 1:100 dilution) was performed according to the instruction of VectaStain Universal ABC kit. Antigenic peptide (VSNAYLYRKRIQP) was used to block CYB561D2 antibody. The staining results was analyzed double-blindly by Quickscore method [17].

### Statistical analysis

Statistical analysis was performed using GraphPad Prism software. All data from cell lines were presented as mean ± SD and statistical analysis was performed by two-tailed Student t test for two groups and one way ANOVA with Newman–Keuls post hoc test for more than two groups. All data from clinical samples were presented as whiskers-box plots and non-parametric Mann–Whitney U-test was used for two groups and Kruskal–Wallis test followed by post hoc Dunn’s multiple comparison test was used for more than two groups. Analysis of expression data and survival data in REMBRANDT and TCGA datasets was performed as previously described [18]. Statistically significant differences were defined as P < 0.05. For all, *P < 0.05, **P < 0.01, ***P < 0.001.

## Results

### CYB561D2 expression is up-regulated in gliomas

**Fig. 1:**
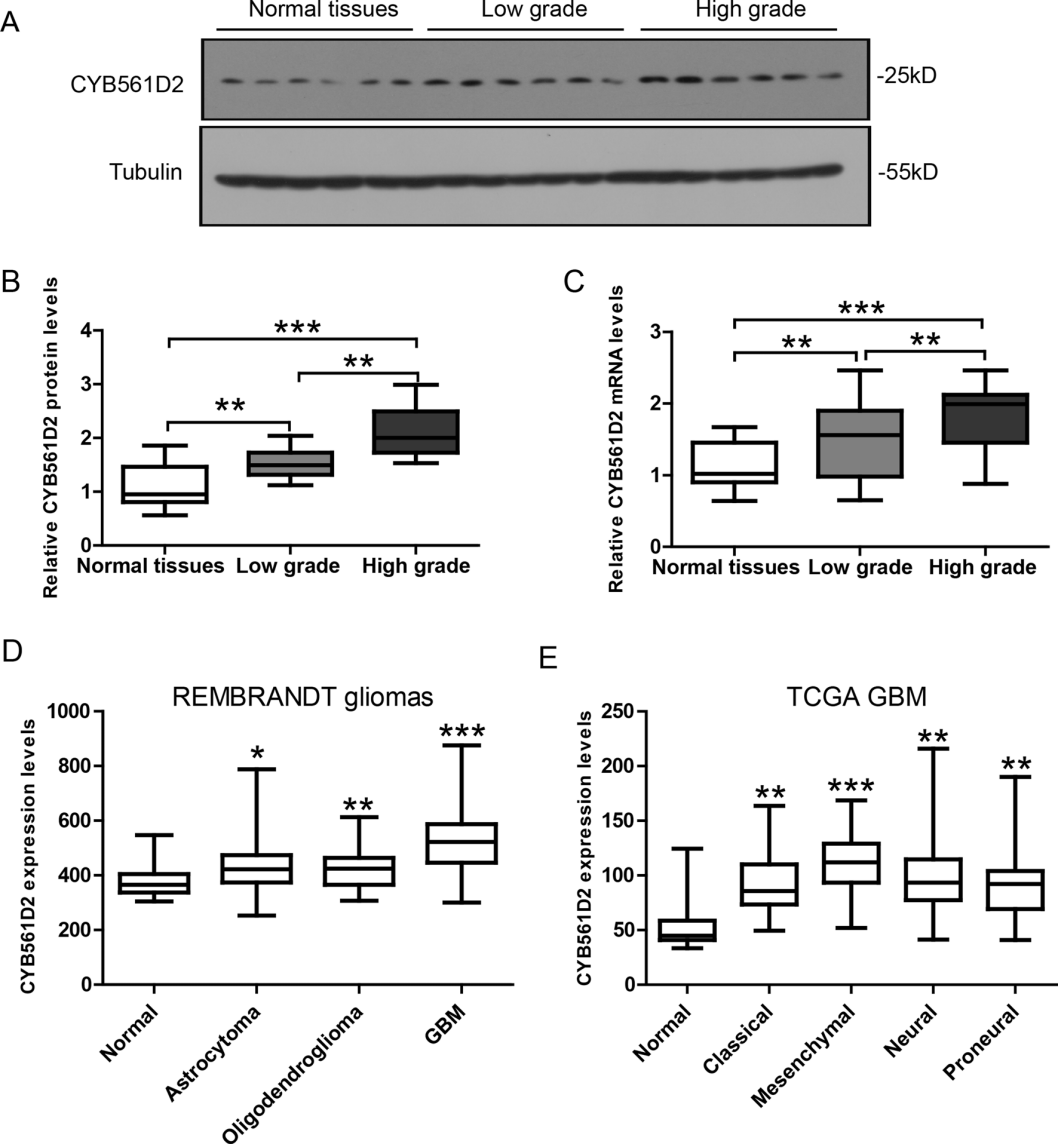
CYB561D2 expression is up-regulated in gliomas. **A** Representative western blot and **B** its quantification showing CYB561D2 protein levels in normal brain tissues (n = 7), low-grade gliomas (n = 14) and high-grade gliomas (n = 21). **C** qRT-PCR results showing CYB561D2 mRNA levels in the same samples. **D** CYB561D2 expression data in REMBRANDT glioma dataset (total n = 524) and **E** TCGA GBM dataset (total n = 454). Data were presented as whiskers-box plots. For all, *P < 0.05; **P < 0.01; ***P < 0.001

CYB561D2 expression levels in gliomas (n = 35) and control tissues (n = 7) were detected by western blot and qPCR. Western blot (Fig. 1A) and its quantification (Fig. 1B) show that CYB561D2 protein levels were increased in gliomas compared to control tissues, and were further increased in high-grade gliomas. Similarly, qPCR result shows that CYB561D2 mRNA levels were also increased in gliomas compared to control tissues, and were further increased in high-grade gliomas (Fig. 1C). To cross-validate CYB561D2 up-regulation, we explored expression data from REMBRANDT glioma [19] (total n = 524, Fig. 1D) and TCGA GBM (total n = 454, Fig. 1E) datasets. Both datasets show a robust up-regulation of CYB561D2 in gliomas compared to control tissues.

**Fig. 2:**
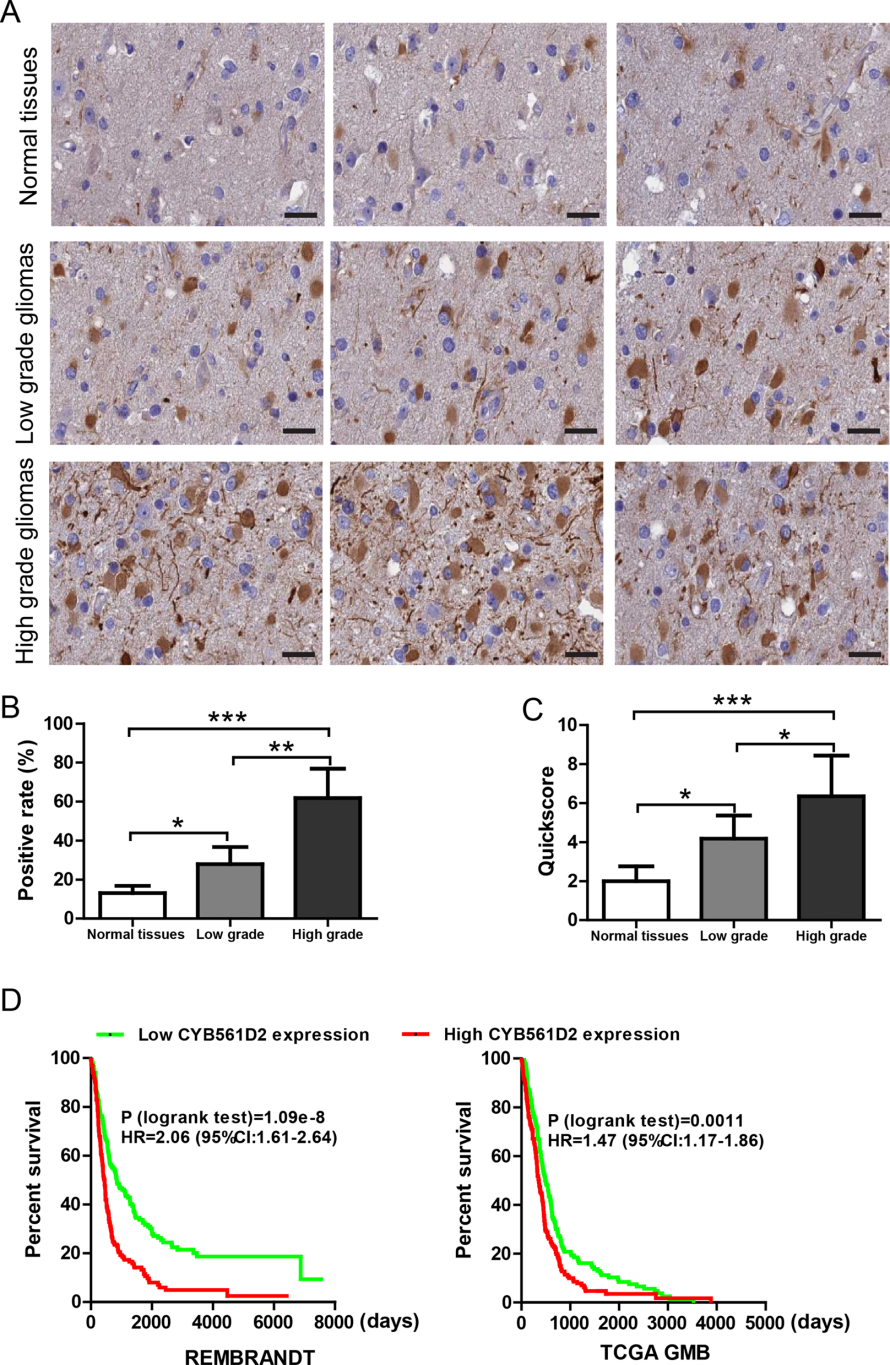
CYB561D2 immunostaining is increased in gliomas. **A** Representative images of CYB561D2 staining in normal tissues, low-grade and high-grade gliomas. Scar bar = 50 µm. **B** Quantification of CYB561D2 staining by positive rate in normal tissues, low-grade and high-grade gliomas. **C** Quantification of CYB561D2 staining by Quickscore in normal tissues, low-grade and high-grade gliomas. Data were presented as mean ± SD. For all, *P < 0.05; **P < 0.01; ***P < 0.001. **D** Kaplan–Meier survival curves of patients classified by CYB561D2 expression in REMBRANDT gliomas dataset (total n = 524, HR = 2.06, P = 1.09e−8) and TCGA GBM dataset (total n = 454, HR = 1.47, P = 0.0011)

CYB561D2 protein level was further investigated by immunohistochemistry in the sections from the same glioma and control samples. Staining glioma sections with CYB561D2 antibody produced clear signal, but replacing CYB561D2 antibody with PBS or blocking CYB561D2 antibody with its antigenic peptide produced no visible signal (Additional file 2: Figure S1). Thus, the CYB561D2 antibody was suitable for IHC experiment. CYB561D2 staining shows a clear cytoplasmic and membranous pattern, mainly in glial cells (Fig. 2A) and the results were further analyzed by Quickscore method. Both the positive rate (Fig. 2B) and the Quickscore (Fig. 2C) were increased in gliomas compared to control tissues, and were further increased in high-grade gliomas.

Kaplan–Meier method was used to explore whether CYB561D2 expression could affect survival of patients in REMBRANDT glioma and TCGA GBM datasets (Fig. 2D). Survival curves of CYB561D2-high and CYB561D2-low expression groups were compared and the results showed that patients with high CYB561D2 expression had significantly shorter survival time than patients with low expression in REMBRANDT gliomas (HR = 2.06, P = 1.09e−8) and TCGA GBM (HR = 1.47, P = 0.0011). Taken together, it suggests that CYB561D2 expression was robustly increased in gliomas and was associated with high histological grade and short survival. Therefore, CYB561D2 may play an important role in the aggression of gliomas.

### *H*_*2*_*O*_*2*_* induces CYB561D2 expression and activates STAT3*

**Fig. 3:**
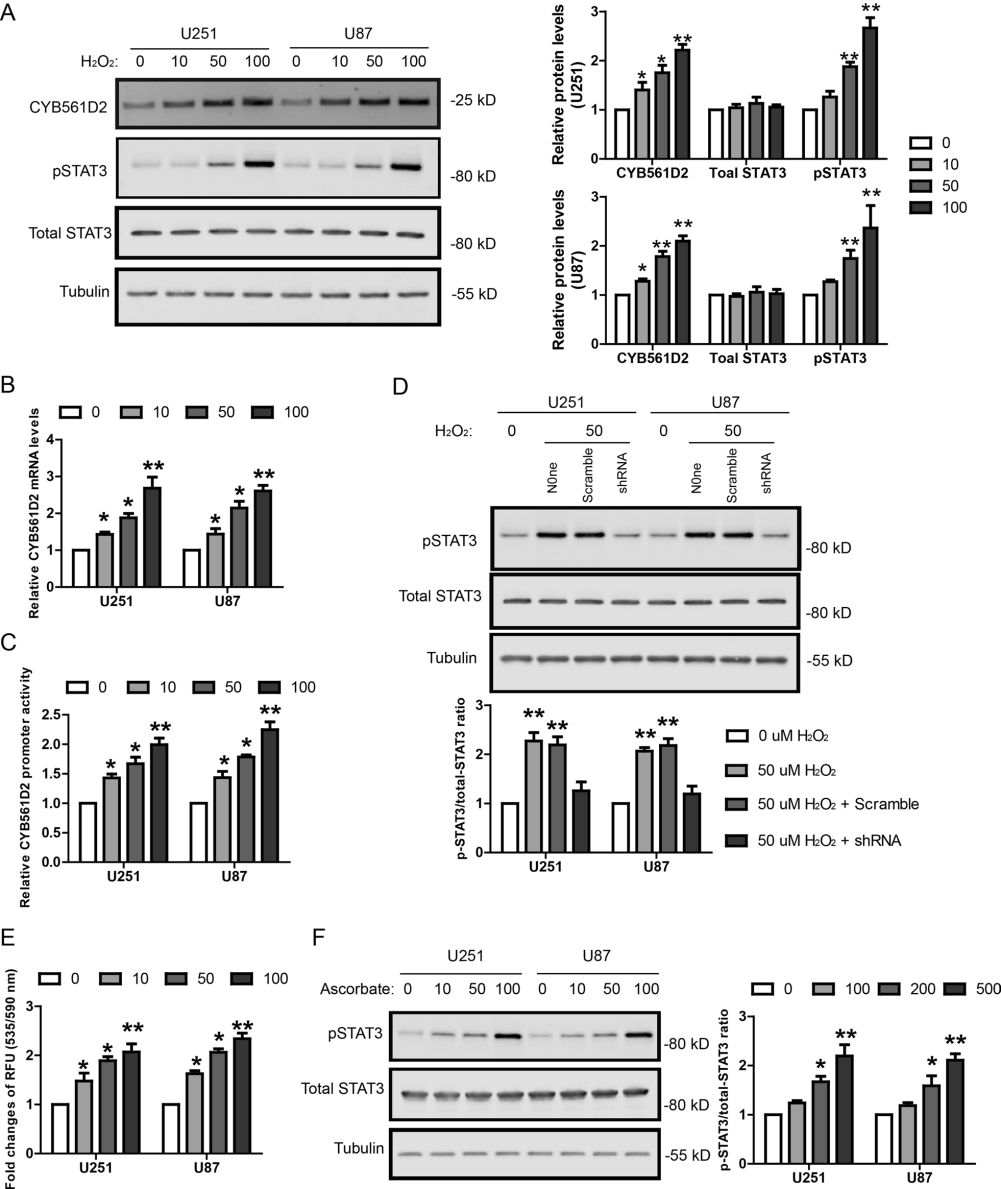
ROS induces CYB561D2 expression and activates STAT3 in gliomas. **A** Representative western blot and its quantification showing the protein levels of CYB561D2, p-STAT3 and total STAT3 in U251 and U87 cell lines treated with H_2_O_2_ at indicated concentrations (0, 10, 50 and 100 µM) for 2 h. **B** qRT-PCR results showing the relative mRNA levels of CYB561D2 in U251 and U87 cell lines treated with H_2_O_2_ at indicated concentrations (0, 10, 50 and 100 µM) for 2 h. **C** Luciferase assay showing the activity of CYB561D2 promoter in U251 and U87 cell lines treated with H_2_O_2_ at indicated concentrations (0, 10, 50 and 100 µM) for 2 h. **D** Representative western blot and its quantification showing the protein levels of pSTAT3 and total STAT3 in U251 and U87 cell lines treated with H_2_O_2_ and infected with Scramble or CYB561D2 shRNA. **E** Quantification of intra-cellular levels of ascorbate in U251 and U87 cell lines treated with H_2_O_2_ at indicated concentrations (0, 10, 50 and 100 µM) for 2 h. **F** Representative western blot and its quantification showing the protein levels of pSTAT3 and total STAT3 in U251 and U87 cell lines treated with ascorbate at indicated concentrations (0, 100, 200 and 500 µM) for 2 h

As increased intra-cellular ROS level is a common feature shared by many types of cancers and CYB561D2 is involved in oxidation–reduction reaction, we investigated whether CYB561D2 up-regulation is resulted from ROS over-accumulation. Indeed, treatment with H_2_O_2_ at indicated concentrations (0, 10, 50 and 100 µM) in glioma cell lines (U251 and U87) for 2 h increased CYB561D2 protein levels dose-dependently (Fig. 3A). In addition, H_2_O_2_ treatment increased pSTAT3 protein levels but not total STAT3 protein levels. This is consistent with previous studies showing that ROS activated STAT3 signaling in various cancers [20–22]. To further confirm that H_2_O_2_ induced CYB561D2 expression, we detected CYB561D2 mRNA level and promoter activity after H_2_O_2_ treatment. Similarly, H_2_O_2_ treatment increased CYB561D2 mRNA level (Fig. 3B) and promoter activity (Fig. 3C) dose-dependently. Taken together, these results suggest that H_2_O_2_ treatment could induce CYB561D2 expression and activate STAT3 in glioma cells.

To investigate whether CYB561D2 mediated the effect of H_2_O_2_ treatment on STAT3, CYB561D2 knock-down was performed by lentiviral shRNA. Cells infected with scramble control or CYB561D2 shRNA were treated with H_2_O_2_ at the concentration of 50 µM for 2 h and STAT3 protein levels were measured by WB. The results showed that CYB561D2 knock-down abolished the effect of H_2_O_2_ treatment on STAT3, suggesting that H_2_O_2_ treatment activated STAT3 through CYB561D2 (Fig. 3D).

As CYB561D2 is essential for the homeostasis of ascorbate, we also measured intra-cellular ascorbate levels in H_2_O_2_ treated glioma cell lines. The results showed that H_2_O_2_ increased intra-cellular ascorbate levels does-dependently (Fig. 3E). It suggests that the H_2_O_2_-induced CYB561D2 has a functional effect on the oxidation–reduction reaction. Moreover, treatment of glioma cell lines with ascorbate at indicated concentrations (0, 100, 200, 500 µM) for 2 h was sufficient to increase pSTAT3 (Fig. 3F). Taken together, these data support that H_2_O_2_ induced CYB561D2 produced more ascorbate which further activated STAT3 in glioma cells. This is consistent with previous studies showing that ascorbate is able to activate diverse kinase pathways such as ERK [23, 24], p. 38; 25, 26 and JAK/STAT [27].

### CYB561D2 up-regulation activates STAT3 in gliomas

**Fig. 4:**
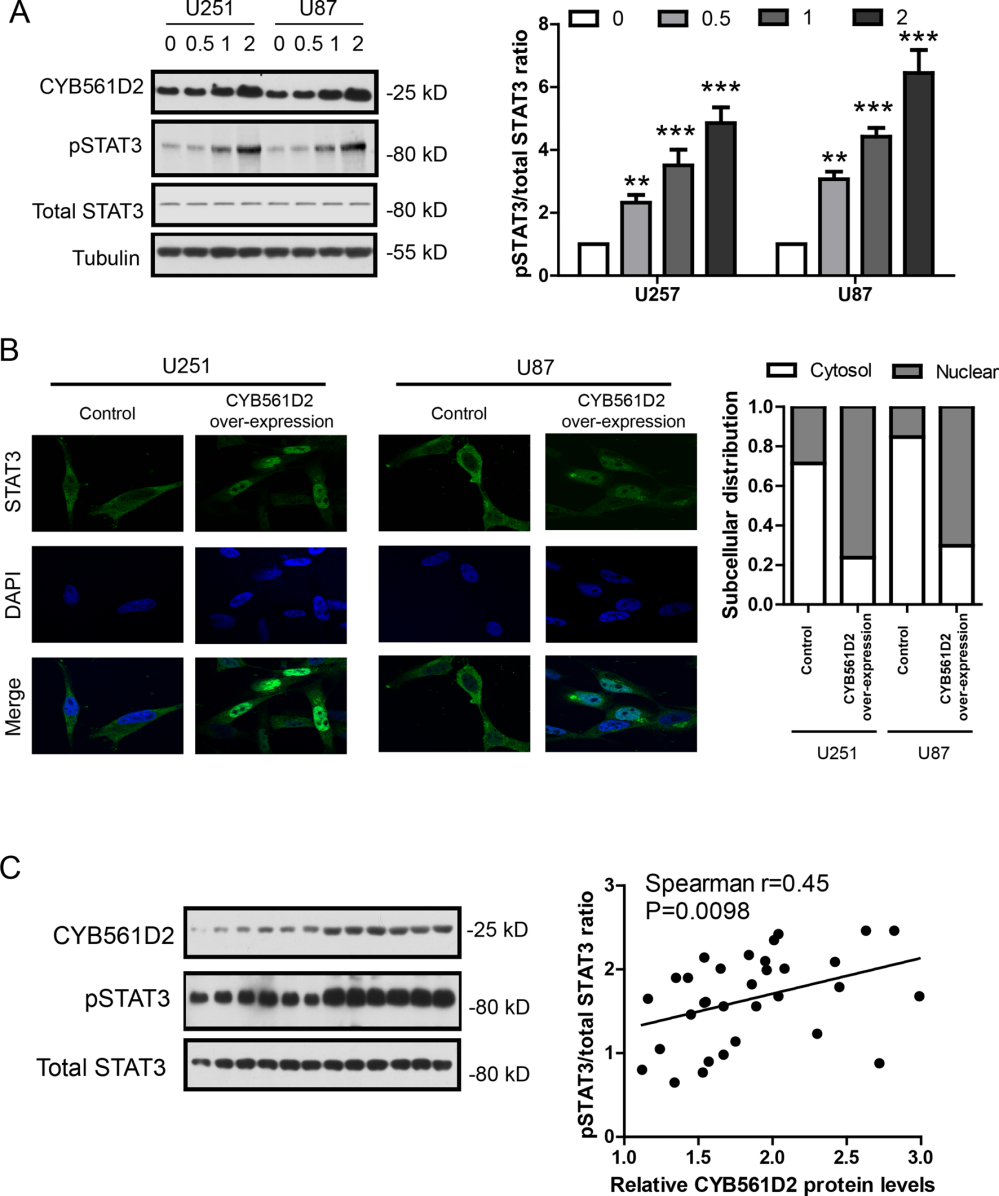
CYB561D2 up-regulation activates STAT3 in gliomas. **A** Representative western blot and its quantification showing the protein levels of CYB561D2, pSTAT3 and total STAT3 after transfection with CYB561D2 plasmid at indicated dose (0, 0.5, 1, 2 μg/well in 6-well plate) in U251 and U87 cell lines. **B** Representative immunofluorescence images and its quantification showing the subcellular distribution of STAT3-GFP after transfection with CYB561D2 plasmid (2 μg/well in 6-well plate) in U251 and U87 cell lines. **C** Left: representative western blot showing the protein levels of CYB561D2, pSTAT3 and total STAT3 in glioma samples. Right: Scatter plot showing the association of CYB561D2 protein level with pSTAT3/total STAT3 ratio (Spearman r = 0.45, P = 0.0098) in glioma samples (n = 35)

To directly demonstrate the effects of CYB561D2 on STAT3, two glioma cell lines were transfected with CYB561D2 plasmid at indicated dose (0, 0.5, 1, 2 μg/well in 6-well plate) for 48 h, respectively. Cell lysates were analyzed by western blot and the results show that CYB561D2 over-expression increased pSTAT3 levels dose-dependently without effects on total STAT3 levels (Fig. 4A). As Phosphorylation of STAT3 would induce its nuclear translocation to modulate target gene expression [28], we also evaluated the effect of CYB561D2 on the subcellular distribution of STAT3-GFP in glioma cell lines. The results show that STAT3-GFP was mainly localized in the cytoplasm in the absence of CYB561D2 over-expression while majority STAT3-GFP was localized in the nucleus in CYB561D2 transfected cells (Fig. 4B). To further support that CYB561D2 activates STAT3 in glioma tissues, we measured STAT3 protein levels in the same samples of gliomas (n = 35) and analyzed its correlation with CYB561D2. The results show that there was a positive association between CYB561D2 and p-STAT3/total STAT3 ratio (Spearman r = 0.45, P = 0.0098, Fig. 4C). Taken together, these results further confirm that CYB561D2 up-regulation activates STAT3 in gliomas and CYB561D2-activated STAT3 might modulate target gene expression to regulate tumor behaviors.

### CYB561D2-activated STAT3 induces the expression of immunosuppressive genes

**Fig. 5:**
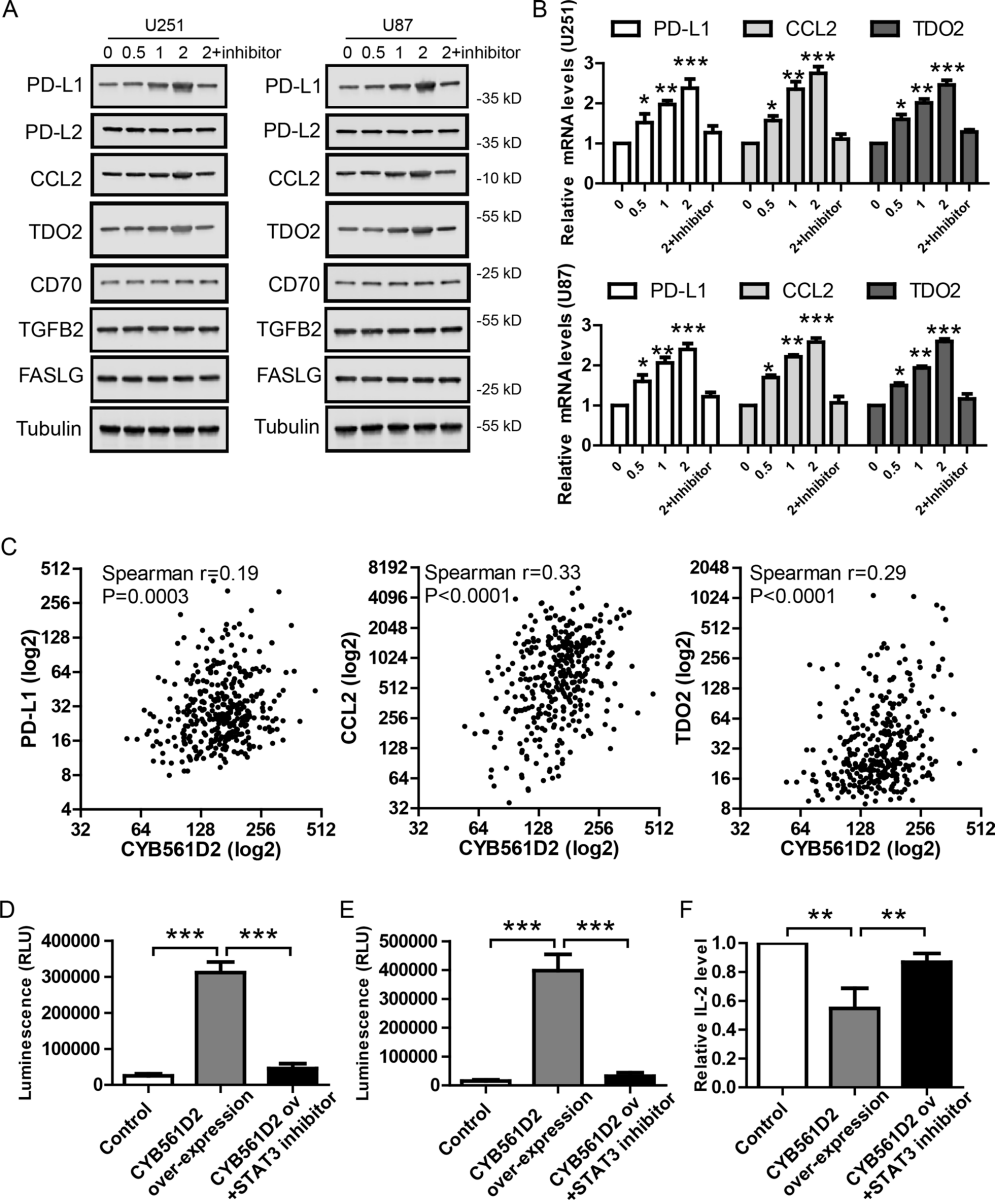
CYB561D2-activated STAT3 induces the expression of immunosuppressive genes. **A** Representative western blots showing the protein levels of FASLG, TGF-β2, CD70, PD-L1, PD-L2, CCL2 and TDO2 after transfection with CYB561D2 plasmid at indicated dose (0, 0.5, 1, 2 μg/well in 6-well plate) in U251 and U87 cell lines. Inhibitor indicates STAT3 inhibitor C188-9. **B** qRT-PCR results showing the relative mRNA levels of PD-L1, CCL2 and TDO2 after transfection with CYB561D2 plasmid at indicated dose in U251 and U87 cell lines. **C** Scatter plots of gene expression showing the association of CYB561D2 with PD-L1 (Spearman r = 0.19, P = 0.0003), CCL2 (Spearman r = 0.33, P < 0.0001) and TDO2 (Spearman r = 0.29, P < 0.0001) in TCGA GMB dataset. **D** RealTime-Glo™ Annexin V Apoptosis assay showing the effect of CYB561D2 over-expressing U251 cells on co-cultured mouse T cells. **E** Caspase-Glo® 3/7 assay showing the effect of CYB561D2 over-expressing U251 cells on co-cultured mouse T cells. **F** IL-2 ELISA showing the effect of CYB561D2 over-expressing U251 cells on co-cultured mouse T cells. For all, *P < 0.05; **P < 0.01; ***P < 0.001

It’s well established that STAT3 activates numerous downstream signaling pathways involved in the pathogenesis of cancers [29]. Here, we focus on the immunosuppressive genes as they are promising drug targets. To investigate whether CYB561D2-activated STAT3 induces the expression of immunosuppressive genes in gliomas, we measured protein expression of FASLG [30], TGF-β2 [31], CD70 [32], PD-L1 [33], PD-L2 [34], CCL2 [35] and TDO2 [36] in U251 and U87 transfected with CYB561D2 plasmid for 48 h. WB results show that the protein levels of PD-L1, CCL2 and TDO2 were increased dose-dependently by CYB561D2 over-expression (Fig. 5A) without effect on the expression of FASLG, TGF-β2, CD70 or PD-L2. The effects of CYB561D2 over-expression on the mRNA levels of PD-L1, CCL2 and TDO2 were also detected by qRT-PCR. Similarly, CYB561D2 over-expression up-regulated the mRNA levels of PD-L1, CCL2 and TDO2 (Fig. 5B). In addition, the above effects of CYB561D2 over-expression were blocked by co-treatment of 100 nM C188-9, a potent STAT3 inhibitor. These results support that CYB561D2 up-regulation induced immunosuppressive gene expression through activating STAT3 in gliomas. To further support the effects of CYB561D2 on PD-L1, CCL2 and TDO2 expression, we analyzed expression data in TCGA GMB dataset (Fig. 5C). Indeed, the results show that CYB561D2 expression was positively correlated with PD-L1 (Spearman r = 0.19, P = 0.0003), CCL2 (Spearman r = 0.33, P < 0.0001) and TDO2 (Spearman r = 0.29, P < 0.0001), respectively. Taken together, these results suggest that CYB561D2 regulates the expression of immunosuppressive genes in glioma cells.

To provide direct evidence that CYB561D2 over-expression in gliomas induces immunosuppression of immune cells, we isolated mouse T cells using Pan T cell isolation kit and the mouse T cells were co-cultured with U251 transfected with control vector or CYB561D2. Then, apoptosis of T cells was measured by two independent assays from Promega. RealTime-Glo™ Annexin V Apoptosis assay detects phosphatidylserine exposure (Fig. 5D) and Caspase-Glo® 3/7 assay detects caspase activity (Fig. 5E). The results show that CYB561D2 over-expressing glioma cells induced more apoptosis in mouse T cells compared to control glioma cells. In addition, this immunosuppressive effect was blocked by STAT3 inhibitor. It’s well established that IL-2 secretion is essential for the activity of T cells. So we also measured IL-2 secretion from co-cultured T cells using ELISA. The result shows that CYB561D2 over-expressing glioma cells inhibited IL-2 secretion of mouse T cells in a STAT3-dependent manner (Fig. 5F). Taken together, these results support that CYB561D2 up-regulation in gliomas could result in immunosuppression of T cells via STAT3.

### CYB561D2 knock-down inhibits aggressive tumor behaviors

**Fig. 6:**
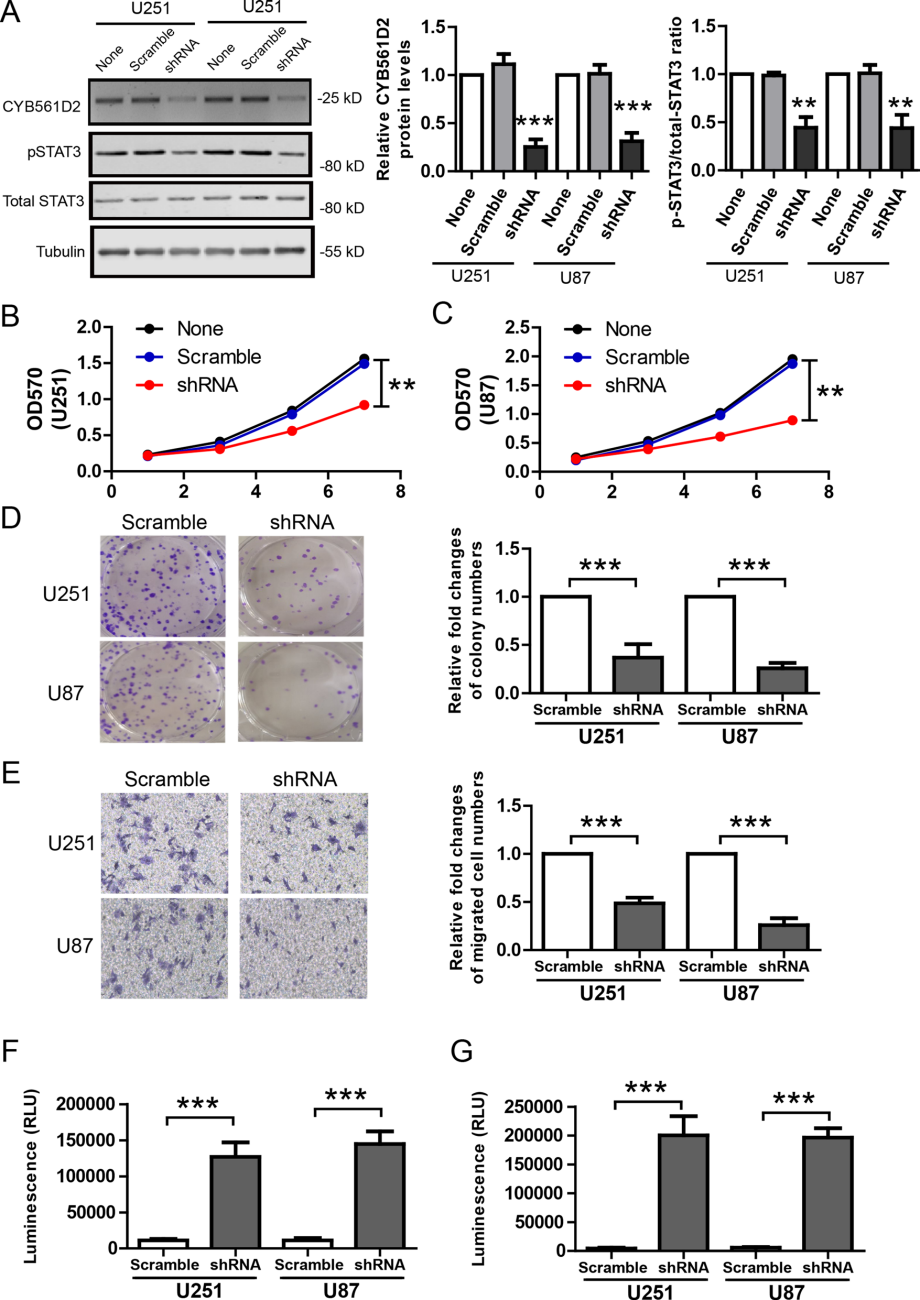
CYB561D2 knock-down inhibits aggressive tumor behaviors in in vitro assays. **A** Representative western blots and its quantification showing the protein levels of CYB561D2, total STAT3and pSTAT3 after CYB561D2 knock-down in U251 and U87 cell lines. MTT assay showing the growth curves of U251 (**B**) and U87 (**C**) after CYB561D2 knock-down. **D** Representative images and its quantification of colony formation of U251 and U87 after CYB561D2 knock-down. **E** Representative images and its quantification of cell migration of U251 and U87 after CYB561D2 knock-down. **F** RealTime-Glo™ Annexin V Apoptosis assay showing the effects of CYB561D2 knock-down in U251 and U87 cell lines. **G** Caspase-Glo® 3/7 assay showing the effects of CYB561D2 knock-down in U251 and U87 cell lines. For all, **P < 0.01; ***P < 0.001

To explore the therapeutic potential of targeting CYB561D2 in gliomas, we used various in vitro assays to measure the effects of CYB561D2 knock-down in U251 and U87 cells infected with CYB561D2 shRNA or scramble at a MOI of 5. WB results show that CYB561D2 shRNA efficiently down-regulated endogenous CYB561D2 protein levels and pSTAT3 levels were reduced at the same time (Fig. 6A). Then, cell proliferation was measured by MTT assay and it shows that CYB561D2 knock-down inhibited cell proliferation in these two cell lines (Fig. 6B, C). In the colony formation assay, CYB561D2 knock-down reduced colony numbers (Fig. 6D). In addition, CYB561D2 knock-down suppressed cell migration (Fig. 6E). Apoptosis was measured by RealTime-Glo™ Annexin V Apoptosis assay (Fig. 6F) and Caspase-Glo® 3/7 assay (Fig. 6G). The results show that CYB561D2 knock-down promoted apoptosis. We also measured the effects of ascorbate on glioma cells with MTT assay. The results show that ascorbate promoted glioma cell proliferation and this effect was largely blocked by STAT3 inhibitor (Additional file 3: Figure S2).

**Fig. 7:**
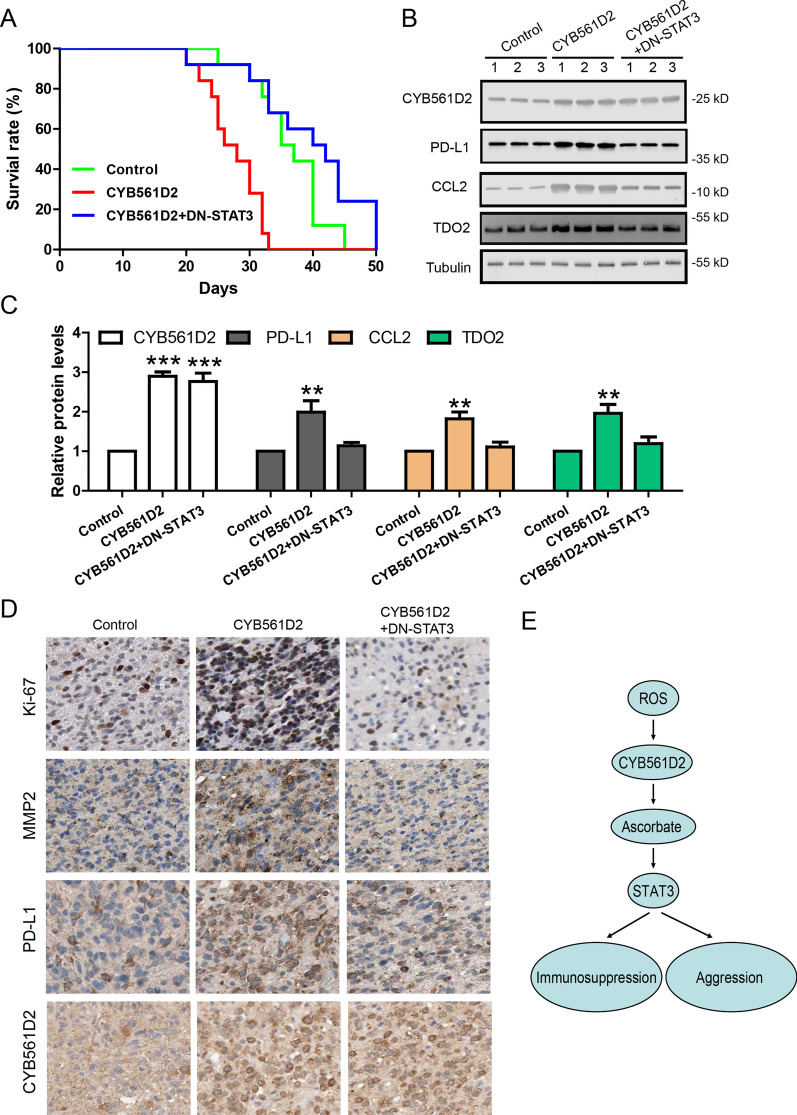
STAT3 mediates the effects of CYB561D2 in in vivo mouse model. **A** Kaplan–Meier survival curves of nude mice bearing intracranial gliomas infected with control, CYB561D2 or CYB561D2 + DN-STAT3. **B** Western blot and its quantification, **C** showing the protein levels of CYB561D2, PD-L1, CCL2 and TDO2 in intracranial tumor tissues. **D** Immunohistochemistry showing CYB561D2, PD-L1, MMP2 and Ki-67 in intracranial tumor tissues. **E** Working model. For all, **P < 0.01; ***P < 0.001

Finally, we established intracranial glioma model in nude mice and mice were randomly assigned to Control group (n = 13), CYB561D2 over-expression group (n = 13) and CYB561D2 over-expression plus dominant negative STAT3 over-expression group (n = 13). Mice bearing CYB561D2 infected cells had shorter survival time compared to mice bearing Control-infected cells (P < 0.01). Mice bearing CYB561D2 plus DN-STAT3 infected cells had longer survival time compared to mice bearing CYB561D2 infected cells (P < 0.001) (Fig. 7A). These results suggest that CYB561D2 enhances in vivo aggression of gliomas and this is consistent with the facts that CYB561D2 up-regulation is correlated with short survival and high histological grade in patients. WB results of intracranial glioma tissues further show that, compared to Control group, the expression of PD-L1, CCL2 and TDO2 was increased in CYB561D2 infected gliomas while co-infection with DN-STAT3 fully rescued this effect (Fig. 7B, C). In addition, immunohistochemistry of intracranial glioma tissues further confirmed that CYB561D2 increased PD-L1, MMP2 (invasion marker) and Ki-67 (proliferation marker) in a STAT3-depedendt manner (Fig. 7D). It suggests that STAT3 mediated the effects of CYB561D2 in in vivo condition.

## Discussion

Taken together, our results support a working model shown in Fig. 7E. Oxidation–reduction reaction is dys-regulated in tumor cells and its balance shifts to over accumulation of intracellular ROS. Although ROS could promote oncogenesis, tumor cells have to respond with increased antioxidant proteins to minimize the potential damage of ROS and make tumor cells more tolerable to high ROS level. As an antioxidant protein, CYB561D2 expression was up-regulated in gliomas by H_2_O_2_. Meanwhile, CYB561D2 and its functional product ascorbate activated STAT3 dose-dependently. In addition, CYB561D2 induced the expression of several immunosuppressive genes like PD-L1, CCL2 and TDO2 in a STAT3-dependent manner. The effect of CYB561D2 on immunosuppression was further confirmed by a co-culture system of glioma cell and T cell. As CYB561D2 expression is correlated with poor prognosis in patients, manipulation of CYB561D2 expression may modulate tumor behaviors. Indeed, CYB561D2 knockdown inhibited aggressive phenotypes in in vitro proliferation, colony formation, migration and apoptosis assays while CYB561D2 over-expression reduced survival rate in in vivo intracranial glioma model. Thus, our results support that CYB561D2 plays an oncogenic role through activating STAT3 in gliomas.

We have noticed that previous studies identified CYB561D2 as a tumor suppressor in lung cancer because of its genetic deletion in some cases and its inhibitory effects on tumor cell growth in certain cell lines. But it’s an open question that whether and how is CYB561D2 involved in cancers beyond lung cancer. To avoid potential bias from small sample size, we cross-validated CYB561D2 up-regulation in two large independent glioma datasets and both show robust up-regulation of CYB561D2 in gliomas. The negative association of CYB561D2 expression with survival was also confirmed in two independent datasets. These robust and reproducible results strongly support that CYB561D2 plays an oncogenic role in gliomas.

A novel finding in our study is the potential association between CYB561D2 and immunosuppressive genes. PD-L1 is PD-1 ligand and their interaction is required for inhibiting T cells [33] and glioma-derived CCL2 recruits regulatory T cells [37]. Tryptophan dioxygenase (TDO2) cleaves tryptophan into kynurenine and the depletion of tryptophan and/or kynurenine accumulation could inactivate T cell [38]. Previous studies have shown that STAT3 activation would further induce numerous downstream target genes including immunosuppressive genes like PD-L1 [39–41] and CCL2 [42]. As we find that CYB561D2 up-regulation activates STAT3, we hypothesized that CYB561D2 up-regulation may increase the expression of immunosuppressive genes through activating STAT3. Indeed, CYB561D2 over-expression increased PD-L1, CCL2 and TDO2 expression, and these effects could be blocked by a potent STAT3 inhibitor. This conclusion is further strengthened by the positive correlation of CYB561D2 expression with PD-L1, CCL2 and TDO2 expression in TCGA GMB datasets. Thus, CYB561D2 might regulate immunosuppressive gene expression to affect the efficacy of immunotherapy in gliomas.

Recent study shows that ROS induces PD-L1 expression in macrophage and results in immunosuppression in breast cancer [43]. However, it’s unclear whether and how could ROS crosstalk with immune checkpoints in tumor cells. Here, we show that CYB561D2, as antioxidant protein that generates ascorbate, was induced by ROS to activate the expression of STAT3 target genes including PD-L1 and lead to immunosuppression in co-culture system. It reveals that CYB561D2 is an important mediator of the crosstalk between ROS and immune checkpoints in gliomas. The crosstalk between H_2_O_2_ and PD-L1 is also independently supported by a recent study showing that H_2_O_2_ leads to up-regulation of PD-L1 in osteosarcoma, non-small cell lung carcinoma and breast cancer cell lines [44]. Thus, combinational targeting of ROS might further optimize the efficacy of immune checkpoint inhibitors in patients with gliomas.

## Conclusion

In summary, we show that CYB561D2 up-regulation induces immunosuppression and aggression via activating STAT3 in gliomas. It reveals that CYB561D2 mediates the crosstalk between ROS and tumor immunity. Thus, targeting CYB561D2 might be a plausible strategy to treat gliomas.

## Supplementary Information

**Additional file 1: Table S1.** Primer sequences.

**Additional file 2: Figure S1.** Validation of CYB561D2 antibody for IHC. Images of CYB561D2 staining in high-grade gliomas with CYB561D2 antibody (left), PBS (middle) or blocked CYB561D2 antibody (right), respectively. Scar bar = 50 µm.

**Additional file 3: Figure S2.** Ascorbate promotes cell proliferation in STAT3-dependent manner. MTT assay showing the proliferation of U251 (left) and U87 (right) cell lines treated with ascorbate in the presence or absence of STAT3 inhibitor.

## Data Availability

All data generated or analysed during this study are included in this published article.
