# Supplementary material for: CYB561D2 up-regulation activates STAT3 to induce immunosuppression and aggression in gliomas
Source: J Transl Med. 2021 Aug 9;19:338. doi: 10.1186/s12967-021-02987-z (PMC8351164; doi:10.1186/s12967-021-02987-z)
Supplement: Supplementary file 1 — Additional file 1: Table S1. Primer sequences. [file 12967_2021_2987_MOESM1_ESM.doc]

| Target gene | Primer sequences |
| --- | --- |
| CYB561D2 forward | TGGCGAAGCTCAAGCTATACC |
| CYB561D2 reverse | CAGGGCATAATACAGCCAGGT |
| PD-L1 forward | TGGCATTTGCTGAACGCATTT |
| PD-L1 reverse | TGCAGCCAGGTCTAATTGTTTT |
| CCL2 forward | CAGCCAGATGCAATCAATGCC |
| CCL2 reverse | TGGAATCCTGAACCCACTTCT |
| TDO2 forward | TCCTCAGGCTATCACTACCTGC |
| TDO2 reverse | ATCTTCGGTATCCAGTGTCGG |
| Actin forward | ACCAACTGGGACGACATGGAGAAA |
| Actin reverse | TAGCACAGCCTGGATAGCAACGTA |
