# Supplementary figures and images for: CYB561D2 up-regulation activates STAT3 to induce immunosuppression and aggression in gliomas
Source: J Transl Med. 2021 Aug 9;19:338. doi: 10.1186/s12967-021-02987-z (PMC8351164; doi:10.1186/s12967-021-02987-z)

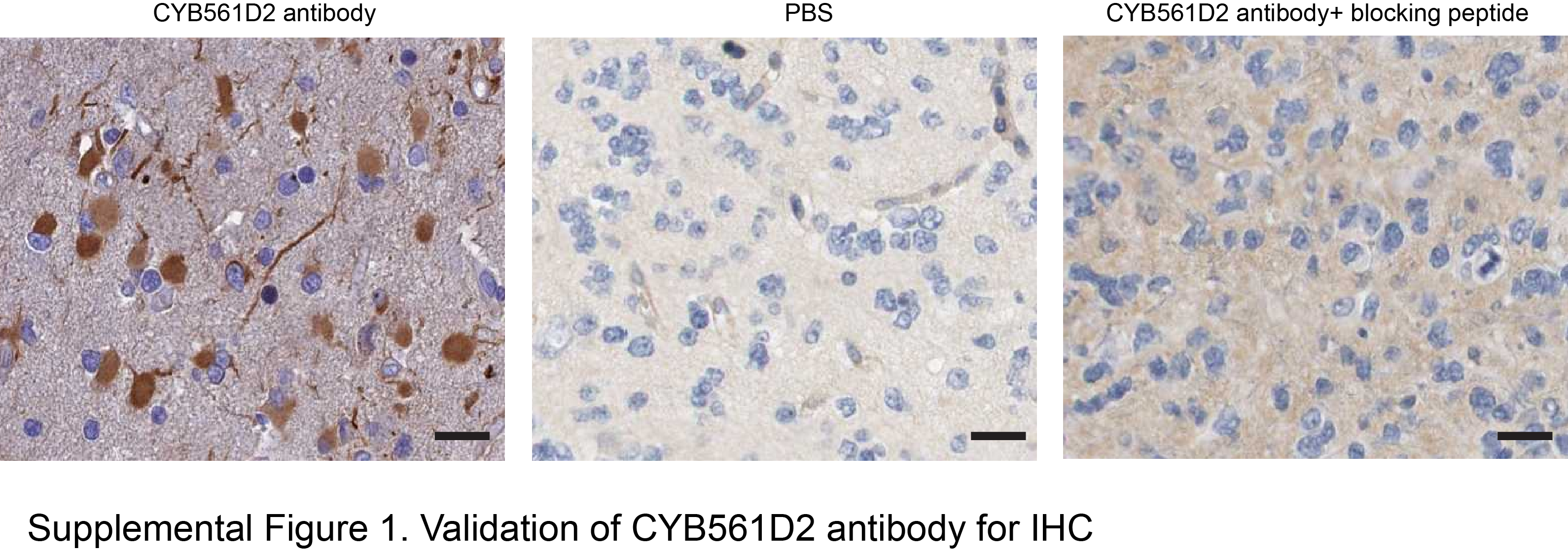

Supplement: Supplementary file 2 — Additional file 2: Figure S1. Validation of CYB561D2 antibody for IHC. Images of CYB561D2 staining in high-grade gliomas with CYB561D2 antibody (left), PBS (middle) or blocked CYB561D2 antibody (right), respectively. Scar bar = 50 µm. [file 12967_2021_2987_MOESM2_ESM.tif]

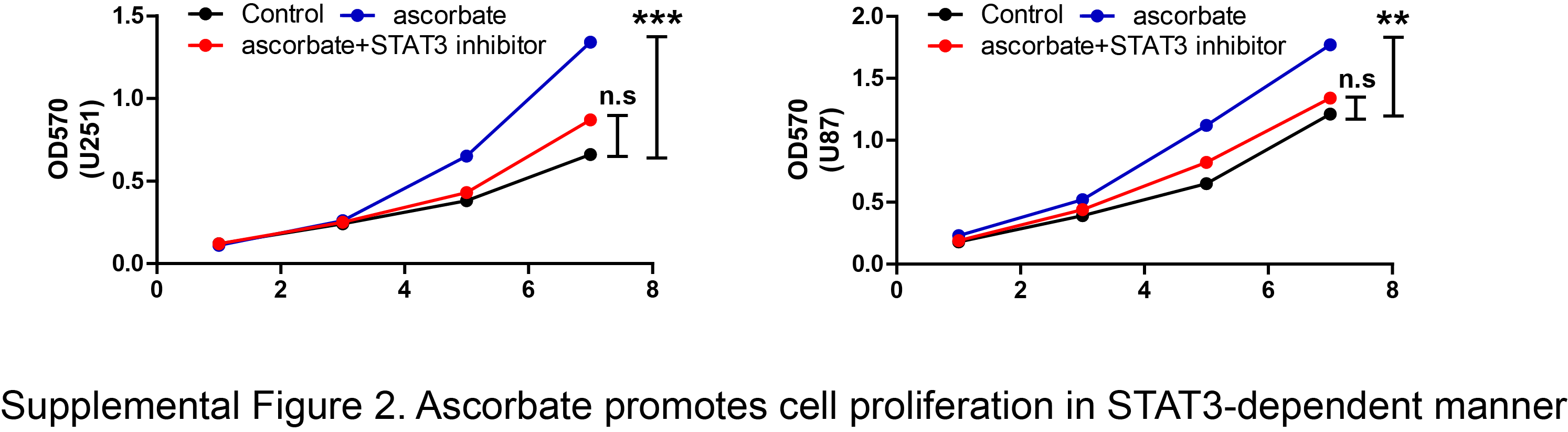

Supplement: Supplementary file 3 — Additional file 3: Figure S2. Ascorbate promotes cell proliferation in STAT3-dependent manner. MTT assay showing the proliferation of U251 (left) and U87 (right) cell lines treated with ascorbate in the presence or absence of STAT3 inhibitor. [file 12967_2021_2987_MOESM3_ESM.tif]
